# Commercializing Sonic Seasoning in Multisensory Offline Experiential Events and Online Tasting Experiences

**DOI:** 10.3389/fpsyg.2021.740354

**Published:** 2021-09-30

**Authors:** Charles Spence, Qian Janice Wang, Felipe Reinoso-Carvalho, Steve Keller

**Affiliations:** ^1^Crossmodal Research Laboratory, University of Oxford, Oxford, United Kingdom; ^2^Department of Food Science, Aarhus University, Aarhus, Denmark; ^3^School of Management, Universidad de los Andes, Bogotá, Colombia; ^4^Studio Resonate | SXM Media, Oakland, CA, United States

**Keywords:** sonic seasoning, crossmodal correspondences, crossmodal pairing, sensory marketing, multisensory experience design, online multisensory tasting, playlist curation

## Abstract

The term “sonic seasoning” refers to the deliberate pairing of sound/music with taste/flavour in order to enhance, or modify, the multisensory tasting experience. Although the recognition that people experience a multitude of crossmodal correspondences between stimuli in the auditory and chemical senses originally emerged from the psychophysics laboratory, the last decade has seen an explosion of interest in the use and application of sonic seasoning research findings, in a range of multisensory experiential events and online offerings. These marketing-led activations have included a variety of different approaches, from curating pre-composed music selections that have the appropriate sonic qualities (such as pitch or timbre), to the composition of bespoke music/soundscapes that match the specific taste/flavour of particular food or beverage products. Moreover, given that our experience of flavour often changes over time and frequently contains multiple distinct elements, there is also scope to more closely match the sonic seasoning to the temporal evolution of the various components (or notes) of the flavour experience. We review a number of case studies of the use of sonic seasoning, highlighting some of the challenges and opportunities associated with the various approaches, and consider the intriguing interplay between physical and digital (online) experiences. Taken together, the various examples reviewed here help to illustrate the growing commercial relevance of sonic seasoning research.

## Introduction

The term “sonic seasoning” refers to the deliberate matching, or pairing, of sound/music with taste/flavour in order to enhance, or modify, the multisensory tasting experience (Spence, 2011b, 2013, 2016, 2017; see also Sedacca, 2016^1^). The realization that consumers experience a multitude of crossmodal correspondences (see Spence, 2011a) between stimuli presented in the auditory and chemical senses originally emerged from the psychophysics laboratory, specifically from the innovative work of Kristan Holt-Hansen (1968, 1976) in Copenhagen, Denmark. Decades later, there has been an explosion of interest in the application of the findings of sonic seasoning research, via a wide range of multisensory experiential events and online marketing activations, particularly with regards to food and/or beverage brands, and their advertising (see Anon, 2012b; Lazarus, 2017). Marketing-led sonic seasoning activations have included everything from curating pre-composed music with the appropriate sonic qualities (such as pitch or timbre; Spence et al., 2013) to the composition of bespoke music/soundscapes designed to match the specific taste/flavour of particular food or beverage products (e.g., Crisinel et al., 2012; Knöferle et al., 2015). One of the earliest examples of this marriage of sonic seasoning and marketing activation involved a bespoke crossmodally congruent music track that was made available to UK consumers online, and that had been designed to match the flavour of the then new Starbucks Via at-home coffee beverage product (see Spence, 2011b).

Given that multisensory flavour experiences typically evolve over time and often contain multiple distinct elements (or notes), there is also an opportunity for researchers and brands to match the sonic seasoning to the temporal evolution of the various elements in the tasting experience. Indeed, a carefully orchestrated piece of music can help the consumer to structure their temporally evolving taste/flavour experience (Crisinel et al., 2013; Wang et al., 2019b). As will be highlighted below, each one of the various approaches to sonic seasoning has their respective strengths and weaknesses in terms of the efficiency, cost of development/implementation, etc. Taken together, the various examples reviewed here help to illustrate the growing commercial relevance of, and interest in, sonic seasoning research (see also Mickiewicz, 2014; Roncero-Menendez, 2015; Spence, 2020a; Spence et al., 2021; Wang et al., 2019c).

According to the definition used here, Tafelmusik (German: literally, ‘‘table-music’’) does not count as sonic seasoning. This is because the music, which was composed with (listening) diners in mind,^2^ was not created specifically to modify, or match, the taste of the meals that were to be eaten while listening to it. Nor, while we are on the topic, would J. S. Bach’s Café Cantata count because, once again, simply composing music on the theme of a particular food or drink product (or flavour) fails to meet the definition of sonic seasoning given above. Closer to meeting the definition would have to be the Italian Futurist’s use of music and environmental sounds paired with specific dishes in the 1930s (see Marinetti, 1932/2014). Other digital and/or online interventions that connect music with food preparation, such as The Concerto App for Häagen Dazs (developed by Goodby, Silverstein and Partners, Inc^3^; Tran, 2013), which showed musicians playing over one’s tub of ice-cream while waiting for it to temper (i.e., soften) when removed from the freezer, or Barilla’s recent release of Spotify playlists that last exactly as long as you should cook pasta (Spary, 2021), also fail to qualify as sonic seasoning for much the same reason.

### Sonic Seasoning, Sensploration, Sensory Marketing, and Synaesthesia

Widespread press interest in the topic of sonic seasoning hints, perhaps, at the somewhat surprising nature of the phenomenon (e.g., Anon, 2012a; Barton, 2012; Eplett, 2013; Hui, 2013; Victor, 2014; Sanderson, 2015; Twilley, 2015; Andrews, 2016; Anson, 2016; McEachran and O’Mahony, 2016; Marston, 2018). It can be considered as a natural extension of the growing interest in the field of sensory marketing (Yeoh and Allan, 2020) and synaesthetic design (Haverkamp, 2014). However, while crossmodal correspondences and sonic seasoning undoubtedly do share a number of similarities with the phenomenon of synaesthesia, there are also some fundamental differences that should be borne in mind (see Deroy and Spence, 2013; Spence, 2013; see also Rudmin and Cappelli, 1983; Crisinel and Spence, 2012b; Sachse-Weinert, 2014; Knapton, 2015; Robson, 2017). For example, the crossmodal correspondences that underpin the authors’ approach to sonic seasoning are shared by the majority of individuals, even extending across cultures when this has been tested explicitly (see Knöferle et al., 2015, for one such cross-cultural demonstration; see also Taitz et al., 2019). By contrast, and by definition (see Grossenbacher and Lovelace, 2001), the specific relations between inducer and concurrent in the case of synaesthesia are idiosyncratic, with different cross-sensory mappings reported in different individuals. Thus, synaesthesia provides little insight into the design of consensually meaningful crossmodal correspondences. It is also worth highlighting here that cases of synaesthesia involving audition and the chemical senses actually turn out to be exceedingly rare (see Day, 2005; see Hänggi et al., 2008, for one of the few examples that have been documented in the literature).

Almost a decade ago now, the beer writer Pete Brown curated a number of live events pairing beer-music in the UK (see Brown, 2012a, b). In this case, the pairings were also largely idiosyncratic, though undoubtedly meaningful to the host (cf. Eschevins et al., 2019). Sometimes the suggested pairing incorporated semantic matching in terms of shared place of origin (e.g., The Velvet Underground and Nico, with Brooklyn Lager). Importantly, however, Brown would sometimes also encourage his audiences to look for the perceptual similarity between beer and matching music (Pixies – Doolittle, with Duvel; see also McDonough, 2018). Some of the other suggested music-beer pairings from Brown are highlighted in Table 1. There are occasional mentions of chefs matching the dishes they serve to the work of specific artists, such as Radiohead (e.g., Abbey-Lambertz, 2014; Ozersky, 2014).

**TABLE 1:** A selection of the idiosyncratic music tracks matched with specific beers by British beer writer/expert Pete Brown offered as part of his entertaining beer-music pairing events.

Artist	Track	Beer
Blondie	Parallel lines	Küppers Kölsch
Stevie Wonder	Songs in the key of life	Stone Cali-Belgique IPA
Miles Davis	Kind of blue	Worthington White Shield
Talk Talk	Spirit of Eden	Bass No 1 Barley Wine
Radiohead	OK computer	Cantillon Rose de Gambrinus
Portishead	Dummy	Saltaire Triple Chocoholic
The Stone Roses	The Stone Roses	Thornbridge Jaipur
Jeff Buckley	Grace	Westvleteren 12
The Beatles	Revolver	Timothy Taylor Landlord
Guns N’ Roses	Appetite for destruction	Stone Arrogant Bastard IPA
Kratfwerk	Trans-Europe express	Asahi
Richard Hawley	Coles’ corner	Guinness
Led Zeppelin	4	Goose Island Bourbon County
Patti Smith	Horses	Orval
Brian Eno	Music for airports	Rochefort 10
The Waterboys	Fisherman’s blues	Deuchars IPA

Over the last few years, sonic seasoning (grounded in the crossmodal correspondences) has been incorporated into a number of multisensory experiential tasting events, building on the growing interest in “sensploration” (e.g., Leow, 2015; Spence, 2019b). The latter term refers to people’s growing fascination with, and openness to, multisensory experiential events, as evidenced by the phenomenal popularity of activations like The Singleton Sensorium (Velasco et al., 2013), or the Tate Sensorium (see Pursey and Lomas, 2018; Spence, 2020e, for reviews). Sensploration has been especially popular amongst younger consumers (e.g., millennials and centennials/GenZ; see Birkner, 2016; Xiong et al., 2016).

Going beyond merely modifying a specific element in the multisensory tasting experience (drawing a taster’s attention to it by listening to the appropriate sonic elements, see Spence, 2019a), there is a suggestion that when the crossmodal stimulus combination works especially well, it may result in the emergence of extraordinary tasting experiences (Holt-Hansen, 1976; Spence, 2020a, b), some explicitly elicited by the sonic ASMR (Autonomous Sensory Meridian Response; cf. Anon, 2017; Barratt et al., 2017; Hopkins, 2017). As a case in point, consider here only the following quote from James John, Director of the Bath (now Bristol) Wine School, speaking to the combination of Mozart’s Laudate dominum, and Chardonnay: *“[…] Just as the sonant complexity is doubled, the gustatory effects of ripe fruit on toasted vanilla explode on the palate and the appreciation of both is taken to an entirely new level”* (quoted in Sachse-Wienert, 2012). Such extraordinary multisensory tasting experiences (see Spence, 2020b, for a number of other examples) helps to address the concerns of the occasional naysayers (often, it has to be said, wine experts), who have, until recently, been sceptical that sonic seasoning was anything more than a harmless novelty (Hunt, 2015), or that it would stand up to scientific scrutiny (Jones, 2012, p. 51; see also White, 2008).

Sonic seasoning is but one approach to the pairing of sensations across the senses (Spence, 2020a). There has long been interest in the role of the semantic qualities of music (such as the impact of distinctively French, German, or Spanish music, or classical as opposed to other styles of popular music) on consumer behavior (e.g., Milliman, 1986; North et al., 1997, 1999; Fiegel, 2013; Beckerman and Gray, 2014; Zellner et al., 2017; De Luca et al., 2019; Fiegel et al., 2019), and, more recently, on multisensory flavour perception and judgments of product quality (see Spence and Wang, 2015a, for a review of the emergence of this approach in the world of wine; and Spence et al., 2019, for a more general overview). At the same time, however, there has also been increasing interest from modernist chefs (e.g., Marinetti, 1932/2014; Spence et al., 2011; Spence and Youssef, 2016; Youssef et al., 2019; see also Leonor et al., 2018), and even an airline, in matching nature sounds to the food they serve (see^4^; Silva, 2019).^5^ In addition, the growing awareness of the potentially positive influence of sonic factors over tasting experiences has taken place in the context of an increased understanding of, and complaints about, the deleterious effects of background noise on multisensory tasting experiences (e.g., Spence, 2015b; Bravo-Moncayo et al., 2020; Freeman, 2021; see Spence, 2014, for a review).

### Review Outline

In this narrative review, we first summarize the emerging body of research on sonic seasoning, addressing some of the explanations for this most surprising of phenomena (Section “Multisensory flavour perception and sonic seasoning: The basics”). Thereafter, in Section “Case studies of sonic seasoning: At the nexus of art, science, and marketing”, we summarize a number of case studies of sonic seasoning in action, highlighting the challenges and opportunities associated with the various approaches that have been implemented by researchers/practitioners to date. We explore how some sonic seasoning has been specifically developed for use in on-premises multisensory experiential events (Spence et al., 2014), while others have been designed for customers to access an online digital experience (Spence, 2011b, 2013, 2016, 2017). Having outlined a number of marketing-led case studies covering a broad range of beverages (e.g., beer, wine, coffee) and foods (e.g., chocolate and cheese), we then highlight the benefits of combining physical and digital activations, what is sometimes referred to as “phygital” (see ‘In a phygital world’, 2018; Mikheev et al., 2021). While the majority of sonic seasoning is pre-recorded and accessed through digital devices (and often online), it has, on occasion been performed live (Spence et al., 2013; Wang and Spence, 2015b). However, it is important to highlight how the live performance setting can fundamentally change the dynamic in terms of where a taster’s attention is focused (with live performance typically demanding/capturing more of a taster’s attention than digital sonic reproduction; Spence et al., 2013; Wang and Spence, 2015a). In Section “Phygital: Experiential events combined with online accessibility,” we highlight the important role played by online sonic seasoning solutions in helping to extend the reach and duration of the consumer’s interest that is often generated initially by multisensory experiential events. Finally, in the Section “Discussion and Conclusion,” we conclude with a number of specific recommendations for future online sonic seasoning activations, highlighting the potential benefits of adding a multisensory component, while keeping in mind the dangers inherent in the commercialization of sonic seasoning.

## Multisensory Flavour Perception and Sonic Seasoning: The Basics

In the half century or so since Holt-Hansen first documented the affinity that his participants exhibited between tastes and tones (Holt-Hansen, 1968, 1976), there has been an explosion of interest in the surprising connections (i.e., crossmodal correspondences) that exist between this particular pair of (often unconnected) senses.

### On the Development of Sonic Seasoning Research

Early studies of sonic seasoning focused specifically on the matching of the frequency of a pure tone with food and beverage items presenting a specific flavour (e.g., Holt-Hansen, 1968, 1976; Rudmin and Cappelli, 1983; and see Reinoso Carvalho et al., 2016b, for a more recent study adopting much the same approach). In the majority of contemporary cases of sonic seasoning, specially composed soundscapes and/or pre-recorded music selections have been designed to match, and hence to emphasize, a specific element in the multisensory tasting experience. These include matching the sonic seasoning with specific taste qualities, such as sweet, sour, bitter, or salty (e.g., Mesz et al., 2011, 2012; Knöferle and Spence, 2012; Knöferle et al., 2015; Guetta and Loui, 2017; Watson and Gunter, 2017; Höchenberger and Ohla, 2019), food textural properties such as creaminess (Reinoso Carvalho et al., 2017; see also Crisinel and Spence, 2011), mouthfeel characteristics such as the body of a red wine (Burzynska et al., 2019), trigeminal qualities such as spiciness (Wang Q. et al., 2017), aromas such as citrus or vanilla (Bronner et al., 2012), and full-blown flavour experiences such as represented by the Cadbury Flavourites activation (see Arrigo, 2017^6^).

The relative timing of sonic seasoning with respect to the related taste/flavour experience also plays an important role in determining the magnitude of any crossmodal effects (see Wang et al., 2020). In particular, the influence of sonic seasoning over taste/flavour perception is more pronounced when the sonic component is presented at the same time as, or prior to, tasting the associated food and/or beverage, suggesting that sound enhances the taste experience via priming sensory expectations and/or drawing one’s attention to specific taste/flavour elements (i.e., rather than as a result of multisensory integration). The crossmodal effects of sonic seasoning dissipate if the sonic element is presented after a participant had finished tasting.

### The Temporal Evolution of Multi-Element Flavour Experiences

**FIGURE 1:**
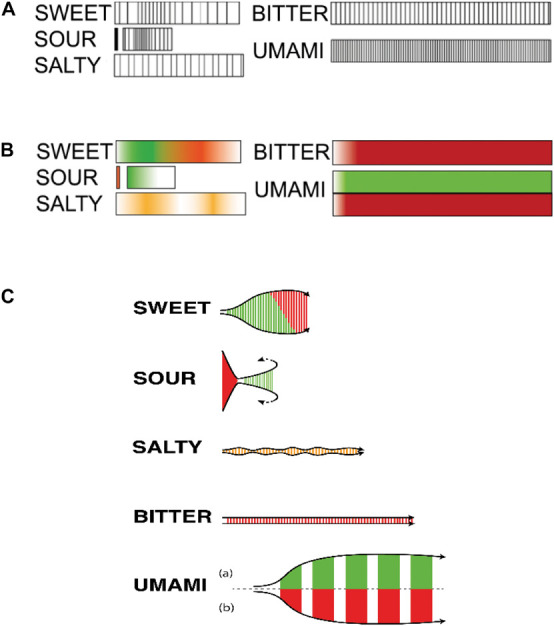
**(A)** Temporal characteristics of taste experiences showing the intensity (thickness of the lines), the movement (frequency of the lines) and the length of the taste experience; **(B)** Affective characteristics of taste experiences (green = pleasant, red = unpleasant, orange = neutral, white = absence of taste). Note that umami was rated as both pleasant and unpleasant; **(C)** All taste characteristics combined: the temporality shown through the length of the schematic; affective reactions through the color (green pleasant, red unpleasant, orange neutral experience); and the embodiment through its form (mouth feeling). [From Obrist et al. (2014), Figures 2, 3, 5].

There is a growing recognition in the food/sensory science community that many food and beverage products deliver a multisensory flavour experience that can perhaps best be described as complex (though see Spence and Wang, 2018a, on the problematic definition of flavour complexity). Many such complex flavour experiences (which often tend to involve fermented flavours; Dunkel et al., 2014; Haagen-Smit, 1952) not only deliver a range of identifiable elements in the flavour experience, but these various notes tend to evolve, develop, and/or change over the course of a tasting experience. Indeed, it turns out that even simple tastants when delivered in water produce a tasting experience that is often dynamic rather than static, with the intensity, and the pleasantness, of the experience varying (i.e., quantitatively) over time (see Obrist et al., 2014; see also Crisinel and Spence, 2012c). Figure 1 shows how even basic tastes, which one might consider the simplest of (taste) stimuli, have temporal profiles that develop predictably over time in both their sensory-discriminative and hedonic dimensions.^7^

Several contemporary sensory science techniques have been developed specifically to help assess the temporal-evolution of such complex multisensory flavour experiences. These include the Temporal Dominance of Sensations (TDS) approach (e.g., Pineau et al., 2012; Charles et al., 2015; Galmarini et al., 2016; Wang et al., 2019b; Higgins et al., 2021), as well as Temporal-Check-All-That-Apply (TCATA; Dooley et al., 2010; Ares and Jaeger, 2013; Wang et al., 2021b; see also Castura, 2020; see also Hyde and Witherly, 1993). The studies that have been published to date using such techniques have revealed the complex temporal evolution of different notes in the multisensory flavour profile of many food and beverage products. Thus far, these techniques have primarily been used for those flavours that are conventionally considered as complex such as coffee and wine (see Spence and Wang, 2018b). It remains an open question as to how many of our everyday taste/flavour experiences exhibit such temporal richness/variation, and/or whether it is something that consumers only notice when they have their attention drawn explicitly to it (e.g., when taking part in such laboratory studies, or as a result of a sonic seasoning experience; see Spence, 2019a).

**FIGURE 2:**
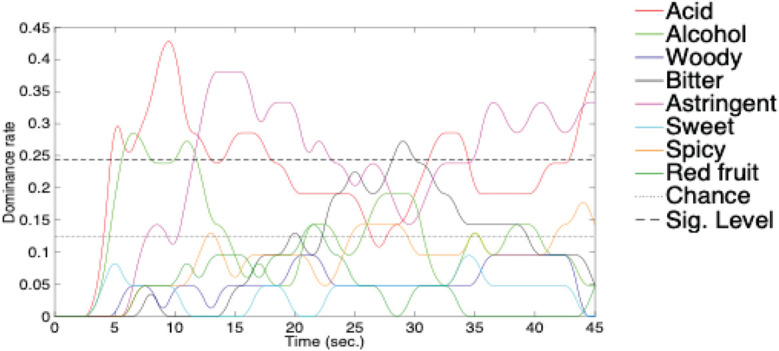
A mouthful of Manos Negras Pinot Noir 2014 held in the mouth for a period of 45 s. Note that the horizontal dashed lines indicate chance level responding (given the eight possible descriptions) and a significant response across the group of participants whenever the time series exceed a dominance ration of 0.25. [From Wang et al. (2019b)].

It might be considered more appropriate to develop soundscapes that evolve over time to match the typical flavour profile than to use music/soundscapes with a uniform association over the whole length of the track (see Spence, 2021b). However, one challenge with such an approach is that it requires a stereotypical tasting regime (to make the tasting experience as uniform and predictable as possible). Indeed, in many of the laboratory studies that have been conducted to date, data collection has occurred while a small group of participants are invited to hold a specific food or beverage product in their mouths for a pre-specified period of time. For instance, Wang and Spence had their participants hold a piece of chocolate in their mouth for 90 s as it slowly melted. Meanwhile, the participants in Wang Q. J. et al.’s (2017) study held a mouthful of Manos Negro wine for 45 s before swallowing, thus allowing the different flavour notes to develop (see Figure 2).

Given the complex nature of so many of our real-world “complex” taste experiences, one might question the appropriateness of delivering sonic seasoning that is itself relatively constant over time, such as music or soundscapes that happen to be associated with a seemingly uniform level of sweetness, say. Yet this is the approach that has been adopted in the majority of sonic seasoning activations that have been published to date (e.g., Wang Q. et al., 2017; Wang et al., 2020). While such solutions undoubtedly have their place in modulating the more constant elements of basic taste, such as sweetness (see Crisinel et al., 2012; Blecken, 2017), there is clearly scope to enrich the temporal matching of sensations in auditory and chemical senses. Bear in mind here only how “common fate” is one of the most important Gestalt grouping principles (Spence, 2015a). Nevertheless, one might also consider the similarities not only in the structure of the tasting experience with a musical excerpt, but the structure of meals and music more generally, as highlighted by Rozin and Rozin (2018); cf. McNeil (1993-1994).

**FIGURE 3:**
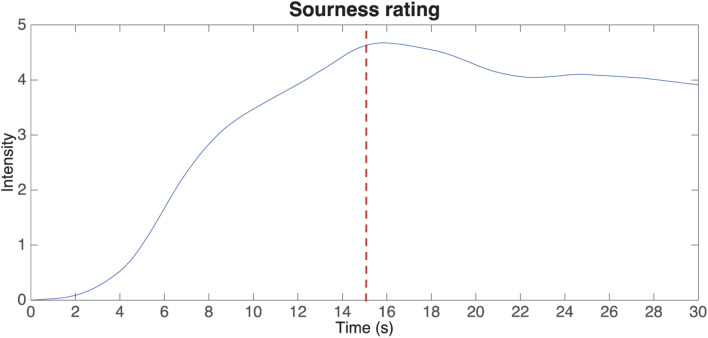
Mean values of time intensity (TI) values of sourness rating over time. The dotted line is shown at the 15-s mark, where the soundtrack changed from “sour” to “sweet.” [From Wang Q. J. et al. (2017)].

One of the other challenges with using pre-composed/pre-recorded music relates to the fact that it can change in its crossmodal associations mid-track, as in the shift between major and minor modalities in Queen’s Bohemian Rhapsody (Crawshaw, 2012; though such examples are perhaps rare). Wang Q. J. et al. (2017) has highlighted the impact of a sudden change of music on the TI response (see Figure 3).

### Sensation Transference

The downside of using specially composed music tracks (or soundscapes) is that they may not offer the same emotional punch as more recognizable popular music tracks (e.g., Reinoso-Carvalho et al., 2020a, b). This is important, given that the latest research from Reinoso-Carvalho and his colleagues suggesting that “sensation transference” effects may be more pronounced than sonic seasoning (see also ‘The delicate connection between sound and taste’, 2021). Sensation transference, or affective ventriloquism (Spence and Gallace, 2011), refers to the fact that our feeling about one stimulus (music in this case) is often transferred to our ratings of another stimulus. However, it would be good to replicate this pattern of results across a wider selection of examples of pre-recorded music tracks and specially composed sonic seasoning soundscapes before making any definitive judgments on this score (if you’ll excuse the pun). In fact, rather than an “either/or” approach, one might consider using both bespoke music and curated popular music within the same experiential activation. Here, it is also worth bearing in mind that emotion provides one basis for sonic seasoning (i.e., sweet = consonant). As such, it is not always easy to tease apart sensation transference from sonic seasoning (Wang and Spence, 2017, 2018).

## Case Studies of Sonic Seasoning: At the Nexus of Art, Science, and Marketing

In this section, we summarize a number of public-facing sonic seasoning activations that have occurred over the last decade. The majority lie at the intersection of art, science, and marketing. While the inspiration for the musical choices has typically come from the results of laboratory research, the creative application of the research findings/recommendations in the design of the experience has not always preserved the underpinning crossmodal correspondences successfully (see Crisinel et al., 2013). As the reader will see, some of these activations have afforded researchers an opportunity to gather data and further our knowledge of crossmodal associations, ultimately adding to our understanding of the extent of, and mechanisms underlying, sonic seasonings. In those instances, we reference relevant published scientific research in the case study description. The majority of these activations, however, draw on applied science to serve a marketing end, often with little thought to scientific validation (see Table 2 for a chronological summary of branded commercial (i.e., public-facing) examples of sonic seasoning. We will discuss the implications of both of these approaches in our conclusions.

**TABLE 2:** Chronological summary of brand-related sonic seasoning activations that have appeared in the public/commercial sphere over the last decade.

Product/brand	Year	On- vs. offline activation	Modalities involved	Auditory stumuli used / dominant sound attributes manipulated and/or stressed	Taste/flavour attributes matched/modified	Comments
Starbucks (UK)	2011	Online	Auditory	Focus on pitch of specially- composed music track	Bitterness	Contemporary instrumental music track composed
Via coffee product						to match taste of new at-home coffee beverage.
Various wines	2012+	Offline	Auditory	Nature sounds and musical sounds	Various attributes of wine	Soundscapes recorded to emphasize tastes/textures in wine.
Courvoisier cognac	2013	Offline	Auditory	Instrument: Harp, strings, piano, etc	Violet flower, candied orange, crème brûlée, coffee, etc.	Soundscapes composed for each of six key aroma in cognac & a composition that combined all six elements.
The Singleton whisky	2013	Offline	Multisensory (Aud., Vis., Olf.)	Nature sounds, semantically-meaningful sounds, & pitch	Grassiness, sweetness, & textured aftertaste	Naturalistic soundscapes composed to accentuate grassiness, sweetness, & woody aftertaste of whisky.
Campo Viejo wine	2014	Offline	Audiovisual	Discontinuity, roughness, sharpness, & consonance	Fruitiness/Freshness	Music composed to bring out sweet vs. sour notes in wine.
Campo Viejo wine	2015	Offline	Auditory	Synaesthetically-inspired sounds	Three different red wines	Synaesthetic soundscapes composed to match three wines.
Sony	2015	Offline	Auditory	Pre-recorded music selections	Various dishes	Sonic seasoning incorporated in dining event to promote loudspeakers.
Bang & Olufsen	2015-2016	Offline	Auditory	Pre-recorded music selections	Various dishes	Sonic seasoning incorporated in dining events to launch products & promote brand.
Bookatable	2016	Offline	Auditory	Pre-recorded music selections	Various dishes	Musical playlists created to match three-course meals at five restaurants.
Cadbury’s Cadbury’s chocolate	2016	Offline	Auditory	Various examples of classical music	Flavoured chocolates	Classical musical selections to match different chocolate flavours.
Stella Artois beer	2016	On- + Offline	Audiovisual	Pitch & instrumentation	Bitterness & sweetness	Music video & song created with two instrumentations of music
						track designed to bring out bitter or sweet notes in the beer.
Chocolate (Brussels)	2016/2017	Offline	Auditory	Various examples of pre-recorded music	Flavoured chocolates	Music playlists to match different tastes/flavours in chocolate from
						three local brands (Leonidas, Frederic Blondeel, Passion Chocolat).
Cadbury”s chocolate	2017	Offline	Auditory	Bespoke music compositions designed as remixable loops, using pitch, articulation, timbre, tempo, & rhythm	Diary Milk, Caramel, Fruit & Nut, Whole Nut, Daim, Oreo, Crunchie Bits, Jelly Popping Candy	Crossmodally congruent soundscapes for eight Cadbury flavoured chocolates, also matched to emotions via a Semantic Differential Tool.
Chivas Regal’s Regal’s Ultis	2017	Offline	Multisensory (Aud., Olf., Tact.)	Various musical parameters including pitch, instrumentation, articulation, timbre, tempo, dynamics, and consonance/dissonance	Five single malt whiskies Citrus, creamy, fruity, spicy, floral	Musical compositions created for each of the five single malt whiskies that together comprised the Ultis blend.
FinnAir	2017	Offline	Auditory	Nature sounds	Various dishes	Soundscapes recorded to capture origins of ingredients used in meals served on Asian longhaul routes.
Glenmorangie whisky	2017	Online	Audiovisual	ASMR-trigger sounds	Three whiskies	Videos created to match three expressions of whisky while sonically-triggering Autonomous Sensory Medirian Response (ASMR).
Chocolate	c. 2018	Offline	Auditory	Various parameters of classical compositions	Four flavoured chocolates	Maxime Goulet plays 4-part classical music to match different chocolates.
Godiva chocolate	2018	Online	Auditory	Pitch, intrumentinstrument, & tempo	Roasted, floral, fruity, creamy, & ‘green’ “green” notes	Soundscape composed whose evolution matched that of chocolate.
Jägermeister	2019	Online	Audiovisual	Four pre-recorded sounds	Bitter, spicy, citrus, and sweet	Tracks composed to bring out different tastes in drink: Bitter, spicy, citrus, & sweet.
Dr. Frank Winery	2019	Offline	Auditory	Pitch, instrument, texture	Oak (i.e., wood/spice notes)	Music composed to match/accentuate oakiness in wines.
Propel isotonic drink	2019	Offline	Audiovisual	Salty: Long decay time, auditory roughness, regular rhythm, minor key, & negative valence	Saltiness and sweetness	Music composed to bring out salty and sweet/fruity notes in drink.
				Sweet: High pitch, connsonantconsonant harmony, slow tempo, legato articulation		
The Glenlivet /	2019	Offline	Auditory	NA	Branded whisky	Sonic whisky tasting events organized in different countries.
The Macallan						
Beck”s beer	2020	On- + Offline	Audiovisual	Pitch	Bitterness/sweetness	Music tracks mixed by DJs with emphasis on pitch-taste correspondence.
Castello cheese	2020	Online	Multisensory	Various auditory parameters	Various flavoured cheeses	Sonic & other sensory elements matched to seven flavoured cheeses.
Café de Colombia	2021	Online	Audiovisual	One pre-recorded medley	Bright-acidity/sweetness, bitterness, aroma	Music paired with videos to accentuate principal flavor notes of Colombian coffee
Keurig coffee	2021	Online	Auditory	Various pre-recorded music	Variety of aroma/taste qualities	Spotify music playlists curated to match taste of five coffee blends.
Magnum ice-cream	2021	Offline	Multisensory	NA	Bitterness/sweetness	Sonic seasoning created to modify taste of Magnum.
Unusual Ingredients	2021	Offline	Auditory	Nature sounds & various sonic qualities including pitch, tempo, & instrumentation	10 different foods/flavours	Soundscapes paired with ten everyday foods availabeavailable as boxed set.

*Note that dining events are not included here, given the lack of strong brand association. See text for further details. Aud. – Auditory; Vis. – Visual; Tact. – Tactile; Olf. – Olfactory.*

### Beer

As has been noted already, the very first studies of sonic seasoning were conducted by Holt-Hansen (1968; 1976) on Carlsberg beer and Carlsberg Elephant lager (the stronger export version). These studies demonstrated that the higher alcohol content export lager tended to be matched with a higher pitch of pure tone, when a small number of participants (c. ten) were invited to select the “pitch of harmony” by varying the sound made by a tone generator. Although a subsequent follow-up only partially supported the claim that different food and beverage products are matched with different pitches of sound (Rudmin and Cappelli, 1983), Reinoso Carvalho et al. (2016b) were able to demonstrate a robust cross-sensory mapping between beers and cola having different pitches. In 2020, Beck’s launched a similar pitch-based experiential activation using music tracks mixed by Brazilian DJs, entitled “Beck’s Frequency” (Fernández, 2020; Gianatasio, 2020b). The pitch of the tracks was adjusted to amplify or suppress the perception of the beer’s bitterness (low-frequency versions around 73 Hz enhanced bitterness, while higher frequency versions around 1046 Hz produced the opposite effect). This endeavor won the brand and their agency, AKQA Brazil, a prestigious Gold Lion and Silver Lion at the 2021 Cannes International Festival of Creativity (AKQA, 2021). At the same time, however, one might also note how 1046Hz is still a relatively low frequency in the context of the range of normal hearing being in the range of 20Hz-20kHz, perhaps suggesting that it is relative rather than absolute pitch that is key (see Spence, 2019c, for a review; and see Parise et al., 2014, on the different pitch-based correspondences observed as a function of the frequency range presented).

### Wine

The matching of flavour with music has been most extensively studied/discussed in the case of wine (see Spence and Wang, 2015a,b,c, for reviews). For example, Spence et al. (2013) had their participants rate the degree to which various pieces of classical music matched fine wines; the best-rated wine-music pairings enhanced wine pleasantness by approximately 10% as compared to tasting the wine in silence (see also Spence, 2020c). The belief that congruent music can enhance the wine tasting experience can be seen in winery-sponsored events such as Krug’s orchestral concerts (King, 2014b) or Campo Viejo’s Streets of London festival featuring matching music written by synaesthetic composer Nick Ryan (Knapton, 2015). It is interesting to note how classical music has often been chosen to pair with wine, perhaps because of its association with class and sophistication (King, 2014a, b; see also Baral, 2015; De Luca et al., 2019; though see also North, 2012). Moreover, the matching between wine and music might best be explained by emotional dominance, or the sense of power, shared between the wine and music (Wang and Spence, 2017).

Beyond matching, music has also been used in artistic and commercial ways to alter the taste of wine (Spence and Wang, 2015b). Wine writer, researcher, and sound artist Jo Burzynska launched the *Oenosthesia* project in 2012, where soundscapes incorporating recordings from the winemaking process were created to emphasize different tastes and textures in various wines (Burzynska, 2012). Meanwhile, as part of the ‘‘Krug Echoes’’ project, artist and composer Henry Ozark created a number of bespoke musical compositions to match different Krug champagnes, such as Krug Clos du Mesnil 2004, Krug, 2004, and Krug 2006 (see^8^; see also Pilley, 2021). The installation has since been presented in Italy, Australia, United Kingdom, and New Zealand (Burzynska, 2018). From a commercial perspective, Dr. Frank winery in the Finger Lakes region featured a soundtrack designed to bring out oak-related elements in the wine’s flavour as a part of a series of wine tasting and tour experiences for their wine club members (Wang et al., 2019a). Those who signed up for the experience were given an explanation of the process of oak maturation, after which they tasted four oaked wines with and without music. The experience was positively received by those who took part, pointing to music-wine tasting experiences as a way for wineries to differentiate themselves from the competition.

### Spirits and Liquors

“The Singleton Sensorium” took place in London, in 2013 (Velasco et al., 2013). Three rooms were decorated in very different styles: One room aimed to recreate an English summer day, another was designed to prime nations of sweetness, while the third room had a distinctly woody theme. Atmospheric soundscapes were also created to play in the background in each of the rooms. The sweet room was decorated in a pinkish-red hue, chosen because that is the color that most people generally associate with sweetness. There was nothing angular in the room; everything was round (the pouf, the table, even the floor plan, and the window frames) because the research shows that people associate rounder shapes with sweetness. There was also the sweet-smelling but non-food-related ambient fragrance and the high-pitched tinkling of what sounded like wind chimes coming from a ceiling-mounted loudspeaker. The latter choice was again based on laboratory research showing that people associate such sounds with sweetness (Crisinel and Spence, 2010; Wang et al., 2015). Every sensory cue had been selected on the basis of the latest research to help prime, consciously or otherwise, notions of sweetness on the palate.

The first room, by contrast, had been designed to prime grassiness on the nose and the sounds of the English countryside in the summer were presented. The final “woody” room was meant to prime a textured finish, or aftertaste, in the mouth and woody sounds were presented (e.g., creaking wood doors, sound of double bass, crackling wood fire, etc.). Over three evenings, nearly 500 people were escorted in groups of 10 to 15 through an experience lasting no more than 15 min. Everyone was given a glass of whisky, a scorecard and a pencil. They filled in one section of the scorecard while standing in each room. They were asked about the grassiness of the whisky on the nose, the sweetness of its taste, and the woody aftertaste. They indicated how much they liked the whisky, and what they thought of the decoration in the room that they were standing in. The grassiness of the nose of the whisky was rated as significantly more intense in the grassy room. The second room brought out the sweetness on the palate (as expected), and the woody room is which people wound up really did accentuate the textured finish of the whisky.

Meanwhile, Courvoisier designed an innovative project where individual instrumental tracks were associated with each of the dominant olfactory notes to be found in a glass of their cognac (e.g., violet flower, candied orange, crème brûlée, coffee, etc.; see Crisinel et al., 2013). A set of six customized scents (Nez de Courvoisier^®^ aroma kit; Courvoisier Import Company, Deerfield, IL United States) was sent to a select group of premier customers, with instructions for the participants to sniff each distinct aroma while listening to the matching instrumental track, after which the lucky customers were to taste the cognac while listening to an additional musical composition which incorporated elements from each of the separate instrumental tracks. The intent of having customers engage in the experience was to help amplify the temporal structure of the music (with different instruments tied to different aroma notes), which, in turn, would enable the consumer to better pick out the various distinctive elements in their tasting experience (see^9^). However, when Crisinel et al. (2013) assessed the consensuality of the matching of individual tracks composed by Laurent Assoulen to correspond to ginger cookies (strings), candied orange (harp), and crème brûlée (piano), the results were not entirely consistent with the composer’s expectations. In particular, participants mostly matched the candied orange to the harp, but they matched the aroma of ginger biscuits to the sound of the piano, while showing no clear pattern for matching sounds to the scent of crème brûlée (see Finn, 2008, for other attempts to compose music specifically to match a fragrance).

Chef Jozef Youssef and author Steve Keller adopted a similar approach as part of a multisensory tasting event celebrating the launch of Chivas Regal’s premium blended whisky, Ultis.^10^ In addition to flavour, tactile, and aroma elements to the activation, Youssef and Keller developed a sonic seasoning accompaniment for each of the five single malt whiskies that together formed the Ultis blend. These soundscapes were designed to reflect not only the flavour profiles of the single malts, but also the regions of the country in which they were distilled. The individual soundscapes were then blended together into a single “opus” that reflected the blending of the malts, with the temporal structure of the opus designed to follow the tasting “notes” that would be experienced as participants sampled a glass of Chivas Ultis. The activations were presented to influencers and members of the press in London, Dubai, Thailand, New York, Turkey, and Vietnam, and was selected as a finalist for the International Sound Awards (see ‘The Sound of Chivas Ultis’, 2017).

Taking a slightly different approach, Glenmorangie commissioned research in order to determine the optimal sensory triggers for ASMR that linked to a Scottish theme appropriate for their whisky (see Barratt et al., 2017). The key triggers identified by this research, including slow-paced close-ups with realistic sound and an absence of background music, were then used by three video artists (Thomas Traum, Julie Weitz, and Studio de Crécy) to create films designed in order to evoke the whisky’s *“terroir, creation, and character”* through ASMR techniques, using “triggers” that relate to whisky and the Highlands in order to elicit emotional reactions (Hopkins, 2017). Intriguingly, while a number of ASMR triggers would seemingly be characterized by higher frequency sounds, low frequency sounds were identified by a number of our respondents as providing effective triggers.

It would seem that whisky brands, in particular, have taken to the use of experiential activations powered by sonic seasoning. A few years after “The Sound of Chivas Ultis,” The Glenlivet conducted its own sonic tasting in India (Vohra, 2019), while The Macallan unveiled its Concept Number 2 whisky at the 2019 TFWA (Tax Free World Association) World Exhibition in Cannes (Turner, 2019) with a sonic whisky tasting. Several other whisky brands are currently working on sonic seasoning interventions, perhaps raising the question of why there hasn’t been similar interest from the gin category, especially given the much publicized international gin revival in recent years.

Elsewhere in the spirits and liquors category, author Felipe Reinoso-Carvalho collaborated in the development of a multichannel activation for Jägermeister in 2019. Named ‘‘Taste Remastered,’’ the activation used sensory stimuli with sonic seasoning in the centre of the multisensory experiences. In this case, different sounds were created by German electronic music composer Sam Sure to amplify the complexity of Jägermeister’s flavour, emphasizing bitter, spicy, citrus, and sweet notes of the liquor.^11^

### Chocolate

The Chocolate Symphony presented at the 2018 IMRF meeting in Toronto provide one example of sonic seasoning for chocolate delivered in a primarily-live-performance setting (see^12^). The composer Goulet typically invites members of the audience at his live concerts (which have been running for a number of years) to accompany each of the four movements of this orchestral suite, with distinct flavours of chocolate, including caramel dark, mint, and coffee-infused, respectively.

Between 2016 and 2017, The city of Brussels (Belgium) also funded a project entitled ‘‘The Sound of Chocolate’’^13^ involving chocolate boxes being sold alongside music playlists that were designed to enhance certain aspects of these chocolate’s taste and flavour. Each playlist paired the brand’s identity of local chocolatiers (Leonidas, Frederic Blondeel, Passion Chocolat), along with sonic seasoning applied to the music of local city artists (Zap Mamma, Duo Aerts, Baikonour). Besides offering a unique way of experiencing Belgian chocolate, the activation also had the place-branding objective of reframing Brussels as a city of innovation.

**FIGURE 4:**
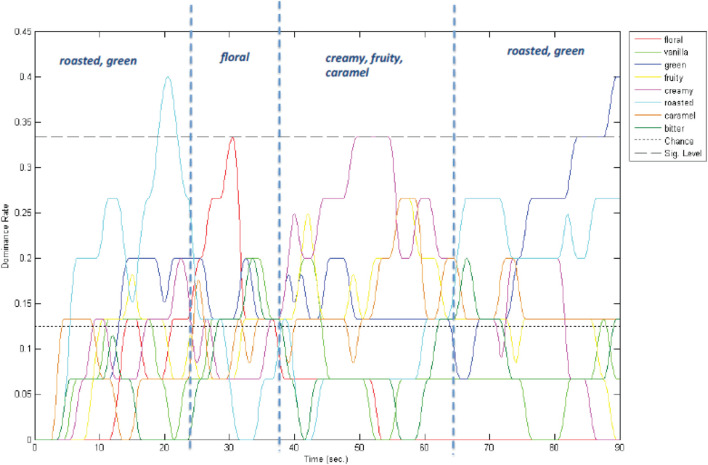
TDS assessment of a piece of chocolate as it melts over the tongue over the course of a minute. [Research conducted by Wang and Spence on behalf of Godiva chocolates].

In a project with Godiva (“A Symphony of Taste”), authors Wang and Spence focused on creating a soundtrack that would, in real-time, unfold with musical elements that corresponded to the flavours in the chocolate. In particular, a TDS study was conducted using one of the company’s dark chocolate to help identify the key taste, flavour, and mouthfeel characteristics that were dominant at any point in time over 90 s, as the chocolate slowly melted in the mouth. As illustrated in Figure 4, the chocolate flavours could be broken down into four phases: 0–25 s featuring roasted flavours; 25--35 s featuring floral notes; 35--65 s featuring fruity flavours and a creamy mouthfeel; and 65--90 s with roasted and ‘‘green’’ flavours lingering on the aftertaste. Wang and Spence then worked with sound designers to create a soundtrack that emphasised the most prominent sensory attributes during each phase of the 90 s soundtrack. The soundtrack began quietly with low-pitched bass (reflecting roasted flavours) then introduced harp and higher pitched synth instruments (corresponding to the floral notes) before opening to a creamy complex crescendo (featuring legato woodwind and brass instruments playing multiple melodies), then slowly fading down to low-pitched bass (reflecting the roasted flavours; see^14, 15^).

In 2016, Cadbury created “The Sound of Flavorites” (The Student Blogger, 2016), a nine-track album featuring bespoke music. The music compositions, recorded by the London Contemporary Orchestra, were based on research commissioned by Cadbury and conducted by neuroscience agency, Mindlab. Low--pitched sounds were found to complement nutty flavours, high pitches complement crunchy texture, steady rhythms complement smooth textures, and up-tempo music complement textures that pop and crackle. In a follow-up campaign the following year, author Keller was commissioned by Cadbury and their agency, Golin, to conduct a series of studies to examine the relationship between eight different Cadbury flavours, a set of corresponding emotions, and a series of original musical soundscapes. The results of the research determined which Cadbury chocolate flavours and matching soundtracks were best suited to a particular emotional descriptor. Based on the outcomes, an experiential marketing event was designed to blend science with performance art, where media and contest winners worked with celebrity DJ Marvin Humes to create personal remixes based on sonic seasonings that matched both an emotional profile and a particular flavour of Cadbury chocolate (see^16^ for more regarding the research, including a case study video; examples of participant created remixes can be found at^17^).

In closing, one might also want to mention the Magnum Pleasure Sensorium, an activation that launched in July, 2021. The activation includes a sonic seasoning element and was co-curated with Food Artist and Anthropologist, Caroline Hobkinson and sound experts Unusual Ingredients.^18, 19^

### Coffee

As mentioned earlier, one of the first commercial examples of sonic seasoning in an online setting involved Starbucks Via coffee, which was launched in the United Kingdom a little over a decade ago (see Spence, 2021a, b). This was the first coffee product designed for the home environment. Given how important ‘the experience’ is to Starbucks (see Spence and Carvalho, 2020), it was important to try and optimize the at-home drinking experience with this activation. Crisinel and Spence (2010, 2012a), assessed the crossmodal correspondences with a variety of coffee aromas, from La New du Café kit.^20^ The resulting recommendations (i.e., crossmodal correspondences with sound) were used as inspiration for a predominantly low-pitched instrumental contemporary music composition by a German composer.

Spence and Reinoso-Carvalho recently collaborated on a project with Keurig coffee in the States. This launch involved using sonic seasoning to curate Spotify playlists that would match with specific coffee blends prepared by this brand. As a result of this collaboration, in early 2021 Keurig released the K-Supreme Playlists — a set of five Spotify playlists designed to enhance the distinctive flavour profiles of five of its coffees (DiPalma, 2021^21^). The Spotify playlist incorporated elements of sonic seasoning as well as semantic matching to bring out specific national identities – i.e., distinctively Colombian music for Colombian coffee mix.

Meanwhile, Reinoso-Carvalho worked with Café de Colombia on a sonic seasoning project that had the main objective of elevating Colombian Coffee beyond its taste (Newsroom, 2021). For its 60 years brand anniversary, Café de Colombia wanted to catch the attention of younger consumers located in high-income countries toward experiencing Colombian coffee in a different way. For this, they decided to rely on sonic seasoning while producing music that enhanced the most important flavour notes of this type of coffee (bright acidity/sweetness, bitterness, and intense aroma). The result was a video campaign that accumulated some 1.5 million views,^22^ and where the music was paired with the visuals, while presented in the form of a sonic seasoning medley, starting with triggering tropical sensations, passing through sweetness, then bitterness, and finishing with a blend of all of these elements. One of the interesting things about this campaign was the fact that coffee baristas were deeply involved in the creative process, since they provided the necessary information on the most distinctive flavour notes of Colombian coffee, and further validated the effectiveness of this sonic seasoning exercise.^23^

### Cheese

Castello cheese (owned by Arla Foods) also recently commissioned a project to illustrate six of their cheeses by means of the crossmodal correspondences (with sounds, shapes, textures, colors, etc.). Working together with Spence, material scientist Dr. Johnny Drain, chef Josef Youssef, and culinary artist Caroline Hopkinson, a series of short videos were created to explain the underpinning science of sonic seasoning and crossmodal correspondences and how it might be creatively incorporated by the chef/culinary artist (see^24^; Gianatasio, 2020a; Spary, 2020). Note that the use of sonic seasoning to influence the taste of cheese should be distinguished here from the use of music to influence the maturation process.

### Isotonic Drinks

Authors Wang and Keller collaborated on an experiential activation created by Pandora for the vitamin water brand “Propel” (Glosson, 2019). In considering the flavour profile of Propel, Wang and Keller focused on two distinct elements in the taste experience of the drink: the presence of electrolytes (minerals like sodium, potassium, and calcium that carry an electric charge when dissolved in a liquid), which contribute to a flavour experience of “saltiness,” and the use of fruit flavours, resulting in an experience of “sweetness.” While the auditory crossmodal correspondences with sweet tastes have been well-established and appear to be reasonably robust (Wang et al., 2015), salty sonic seasoning has been somewhat harder to establish convincingly. Subsequently, a largescale study (*n* = 1,819 participants) was launched which demonstrated that there are, indeed, robust crossmodal correspondences with “salty soundscapes” (see Wang et al., 2021a). In particular, saltiness was most strongly associated with a long decay time, a high degree of auditory roughness, a regular rhythm, minor key, and negative valence. Ultimately, this research informed the creation of bespoke sweet and salty soundtracks that were featured at an on-site “Sound Boost” sonic tasting activation during a brand sponsored fitness festival that included instructors, exercise studios, celebrity trainers and nutritionists, and musical artists. The sonic seasoning was delivered via an interactive digital interface, installed on iPads and incorporated into kiosks at the event. Attendees donned headphones, drank Propel, and moved a slider on the digital interface that allowed them to transition smoothly between the salty and sweet soundscapes. In addition to the sonic stimulus, visual sensory hacks were incorporated into the graphics of the user interface to enhance the crossmodal effects: as participants moved the fader to the salty soundtrack, the colors would desaturate (suggesting a crossmodal correspondence between whiteness and saltiness), and when moving the fader to the sweet soundtrack, the colors would become more vivid (suggesting a crossmodal correspondence between bright colors and sweetness).

### Multisensory Dining Events

GQ Magazine consulted with Wang and Spence for a dinner held in Mexico in 2014, where the suggested music pairings were available on the Deezer platform.^25^ The menu items, including rationales for music pairings, are highlighted in the annotated menu (see Menu 1) shown below. Such pairing based on sonic seasoning can be contrasted with the menus paired with music artists/albums mentioned earlier. Meanwhile at the end of 2016, Spence worked with the online restaurant booking platform Bookatable (shortly prior to it being bought by The Michelin Guide) to curate the musical selections for a festival exploring the links between food and music (Thompson, 2016). For a limited time, a selection of musical tracks were chosen in collaboration with Swedish music producer Axel Bowman match three-course set menus being offered at five established London restaurants [Cigalon, Heliot Steak House (The Hippodrome Casino), Hilton Double Tree, and Marco Pierre White’s Wheeler’s Oyster Bar and Grill Room].

**Menu 1.**

Annotated menu with suggested pre-composed music choices (from authors Wang and Spence) to match the dishes served at a curated sonic seasoning dinner in Mexico in 2014 on behalf of GQ magazine. This dinner was one of the first occasions on which sonic seasoning was used to accompany a meal. The online playlist was made available on the Deezer platform (see http://www.deezer.com/playlist/1054944451).

**Pismo clam cocktail**

*Fresh Clam with acid tones and a spicy touch*

Music pairing:

Marron 5 – One More Night

Emiliana Torrini – Jungle Drum

Explanation: According to research from the Crossmodal Research Laboratory and elsewhere, people tend to associate sourness with music with fast tempo, high pitch, and staccato articulation. The songs were chosen for their upbeat character to bring out the bright, citrus flavours in the dish.

**Octopus and pork belly pozole**

*Pickled and smoked flavours. With Corncob grains and hardened vegetables.*

Music pairing:

Tom Waits – I Hope That I Don’t Fall in Love With You

Tom Waits – Old Shoes [and Picture Postcards]

Explanation: Smoke is associated with low pitch, and what’s more smoky than Tom Waits’ voice? The high pitched, slightly dissonant guitars provide the pickled (sour) accompaniment.

**Dry noodle, quail egg and shrimp crackling**

*The noodle is cooked in tomato sauce with a touch of selfish provided by the shrimp crackling. It’s accompanied with ranch cream, avocado and salty cotija cheese.*

Music pairing:

Julian Gorus – Liszt No. 3 Canzonetta de Salvator Rosa

Phoenix – Lisztomania

Explanation: This dish is savoury and crunchy. Savoury is characterised by very staccato articulation and average pitch. Liszt’s Canzonetta is a very fun, rhythmic song that pairs well with salty, crunchy foods. The second song, Phoenix’s Lisztomania (not a pun on the first song), is higher pitched and faster, which brings out the acidity of the tomato sauce while keeping the salty association going with its staccato rhythm.

**Risotto nero**

*Slowly cooked rice in squid ink. Predominant squid flavour.*

Music pairing:

Francois Salque – Faure’s Elegie, Op. 24

Francois Salque – Faure’s Cello Sonata No. 2 in G minor, Movement II Andante

Explanation: This is a creamy dish with intense flavour and dark color. Low pitch is typically associated with dark colors and deep flavours, so the low voice of the cello was chosen for this dish. In addition, the smoothness of the cello tonality matches the creamy texture of the risotto.

**Milk Rock Cornish au jus with sweet potato puree, shiitakes and french runner beans**

*Rosemary and fresh salvia (sage) flavours. Buttered with a touch of garlic.*

Music pairing:

National Philharmonic Orchestra (London) - Beethoven Symphony No. 6 in F Major, Op. 68 “Pastoral”: I. Allegro ma non troppo

National Philharmonic Orchestra (London) - Beethoven Symphony No. 6 in F Major, Op. 68 “Pastoral”: II. Andante molto mosso

Explanation: This is a savoury, rich dish with multiple layers of flavour. Consequently, music with complex orchestration, rich harmonies, and pleasing timbres was chosen to match the dish. The higher strings and fast tempo in the first movement was chosen to bring out the herbal notes in the dish.

**Pumpkin Creme bruleé**

*Vanilla, cinnamon and pumpkin flavours*

Music pairing:

Billie Holiday – Autumn in New York

Lester Young – On the Sunny Side of the Street

Explanation: The crème brûlée is a dish of contradictions – sweet and bitter, smooth and crunchy. The ballad of Billie Holiday combines high-pitched piano notes (which is associated with sweetness) with the lower pitched, plaintive voice of Billie Holiday (slow, low-pitched music is associated bitterness). The subject of the song – autumn – emphasises the autumn flavours of cinnamon and pumpkin in the dish. The second song by Lester Young continues this pattern of contrasts by pairing the smooth, low-pitched tenor saxophone with the higher-pitched, staccato (“crunchy”) piano backing.

Composer and audio designer Ben Houge has designed a series of avant-garde audio-gustatory events that he calls ‘‘food operas.’’ Houge draws upon his experience with video game music design to create aleatoric, event-driven soundtracks that reflect the dish currently enjoyed by the diner, but which, taken together, creates an ever-changing yet coherent sonic environment in the restaurant over multiple courses (see^26^). Houge, together with Jutta Friedrichs, created an interactive musical dish for the 20^th^ anniversary celebration of the two Michelin-starred Mugaritz restaurant outside San Sebastian, Spain, in 2018.^27^ The dish, consisting of a small acorn-shaped bite of ham fat, cheese, and buckwheat, is served enclosed in a bell jar. When the bell jar was lifted, a pressure-sensing device built into the plate triggered the playback of an individualised soundtrack designed to evoke a sense of nostalgia and curiosity. Furthermore, when the diners touched the food itself, the sound playback changed from music-box to referencing the kinds of things you might expect to find in a Christmas cracker, turning the moment of eating into an almost magical experience. See also Derval (2010), pp. 59–61, and Velasco and Obrist (2020), pp. 6–7 for a couple of other examples of meals that have been organized with a matching sonic element.

In May 2015, Spence worked with Bompas and Parr to create a sonic seasoning event for the launch of Sony’s multi-room speakers (see Press Release, 2015a,b). According Becky Barnes, a Press Association multimedia news reporter present at the event held in Dalston, East London: “I particularly enjoyed the quail breast dish, which we ate while listening to low frequency music. It did feel like the high-pitched notes accentuated the sweetness of the sauce.” (quoted in Anon, 2015). Between 2015 and 2016, author Reinoso-Carvalho co-designed a multisensory menu with Belgian chef Wout Bru, and hosted several product-launching events for another high-end electronics brand, Bang and Olufsen.^28^ These exclusive private events had the objective of reflecting on the importance of quality of sound in the lives of their consumers, and relied mostly on sonic seasoning powered by the brand’s audio-visual products.

**FIGURE 5:**
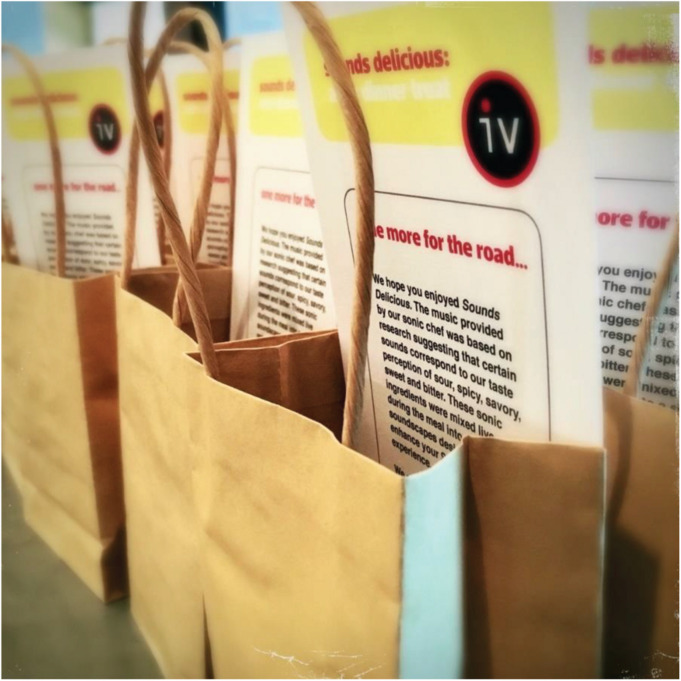
“Sonic doggy bags” delivered to patrons following their multisensory dinner. The cards in the bag contained a URL where diners could download an album of crossmodally congruent soundscapes created on the premises during the meal by a “sonic chef”.

Following their research into the elements comprising the sonic seasoning for spiciness (i.e., piquancy; see again Wang Q. et al., 2017), Keller and Wang devised a multisensory event hosted by chef Deborah Paquette at Etch restaurant in Nashville, Tennessee, in March, 2016. The reservation only, ticketed event for approximately 80 guests featured a four-course meal, with each course concentrating on a specific taste/flavour profile: Sour, spicy, savory, and bittersweet. Sonic seasonings for each course were loaded into Ableton Live (a digital audio workstation), and mixed together during the event by a ‘‘sonic chef’’ into a bespoke impromptu composition as dinners enjoyed each course. Between the courses, white noise was played as something of a ‘‘sonic palate cleanser.’’ In addition to the unique presence and participation of a sonic chef, the four soundscapes he created from the sonic seasonings were recorded as they were performed, and then posted to a website as the meal was concluding. As diners left the event, they were given a ‘‘sonic doggy bag’’ containing chocolate treats and a special URL^29^ where they could download the crossmodally congruent soundscapes created during the meal, see photographs and ingredients of the four courses from the menu, and download a PDF explaining the science behind the event (see Figure 5).

Chef Jozef Youssef also featured a whisky experience as part of his Gastrophysics Chef’s Table menu (entitled “A Taste of Chivas”), designed to demonstrate the crossmodal correspondences with the chemical senses. The experience included a sonic seasoning, with diners being encouraged to enjoy the taste of a glass of Chivas whisky while listening to soundscapes that had been designed to bring out the textured aftertaste or sweetness of the drink (Chambers, 2015).

Blecken (2017) has reported on a marketing-led activation from the Xin café in Beijing where sweet music was played from movement-activated drinking vessels (cf. Mesz and Tedesco, 2021), with the intent that the effect would allow for less sugar to be added to the drink while keeping the perceived sweetness level constant (see Caul, 1951, on the notion of sugar as a seasoning). However, it should be noted that there is no evidence concerning how long-lasting the effects of sonic seasoning delivered in such a manner might be. Here, one might also consider Bompas and Parr who teamed up with Heinz Baked Beans (‘Musical spoons to go with your Heinz beans’, 2013). The limited edition Bompas and Parr baked beans spoons, available for £57. Each spoon had an MP3 player hidden inside. If you bought one, you wouldn’t hear anything until you put the spoon into your mouth. Then the sound waves would travel via your teeth and jawbone through to your inner ear. The flavour–music combinations in this case including cheddar cheese with a rousing bit of Elgar, fiery chilli with a Latin samba, blues for the BBQ-flavoured beans, and Indian sitar music for the curry-flavoured beans.

**FIGURE 6:**
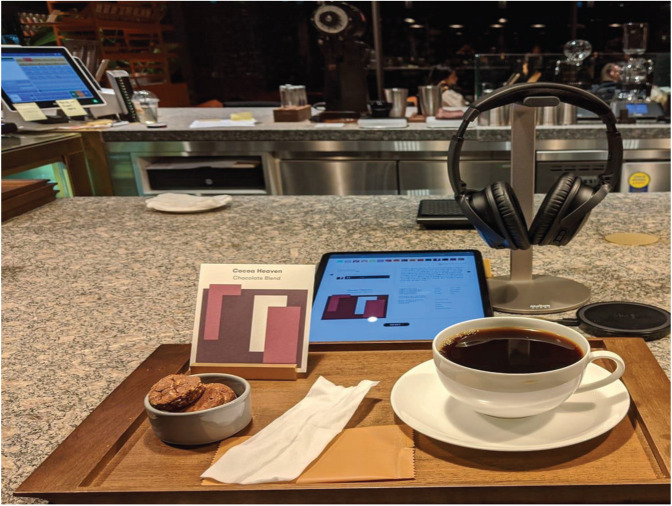
Korean coffee-shop where music is paired with the choice of coffee designed to match the customer’s taste preferences.

Elsewhere, a coffee shop in Korea recently started offering customers musical matches for the specialty coffees that they chose (see Figure 6). Yet, little information is available concerning the principles underpinning the crossmodal pairing in this case (see Spence, 2020a). Nonetheless, this hints at the growing interest in pairing in the wider public sphere, often mediated by digital technology.

Finally, for those wanting to optimize the at-home multisensory dining experience during COVID lockdowns, sonic seasonings can be offered with meal kits, via curated playlists streamed from music platforms like Spotify or Pandora (see Spence et al., 2021, for a review). For example, in February, 2021, chef Jozef Youssef’s Kitchen Theory offered a complete home multisensory experience with Valentine’s Day boxes incorporating a 4-course meal, wine, scented candle and a curated Spotify playlist.^30^ In March, there was a Mother’s Day box with full afternoon tea, glass cake stand, rose atomiser, luxury tea selection and a different Spotify playlist.^31^ While not paired with specific meals, hospitality startup Tock offers a list of playlists from various restaurants, wineries, and bars around the world.^32^

### Interim Summary

The case studies that have been described in this section provide a sense of the commercial interest in, and practical applications of, sonic seasoning. What is striking is how many of the examples involve fermented products (e.g., beer, wine, coffee, chocolate, cheese), perhaps due to the added flavour complexity that fermentation so often delivers (Dunkel et al., 2014), perhaps making these products especially well-suited for sonic sensory activations. Given the themes of this Special Issue, it is also worth noting how many of the examples of sonic seasoning have been made available online.

## Phygital: Experiential Events Combined With Online Accessibility

In principle, there is no reason why sonic seasoning needs to be limited to an online multisensory experience. Yet, the majority of the branded activations reviewed above have incorporated some type of digital user interface (UI) if not offered as part of a multisensory experiential event. The rare exceptions tend to use a physical format for music delivery (e.g., record, cassette, live performance, etc.) idiosyncratically, often to stress the physicality of the medium (see Brennan, 2020; Thompson-Bell et al., in press; cf. Spence, 2020d). A potential drawback to the live performance of sonic seasonings is the possibility that the performance itself shifts the focus of the taster’s attention away from the food or beverage to the performance itself (see Spence et al., 2013). Live performance settings also have obvious size limitations, making them problematic for those brands interested in scaling the experience to include as many potential customers as possible.

Offering online sonic experiences provides a potential solution to this problem of scale. A crucial limitation of experiential tasting events, such as The Singleton Sensorium (Velasco et al., 2013), the Campo Viejo Color lab (Spence et al., 2014; see also Knapton, 2015), or even the multiple Chivas Ultis events (see Section “Spirits and liquors”), is that no matter how large the event itself, only a very limited number of people will ever have the opportunity to attend. What is more, such events tend to be of only short duration. Hence, in order in increase the scale, while at the same time prolonging the tail of online discussion around the event thought digital and media channels, it helps to offer some kind of online version of the experience. In one such phygital offering, Stella Artois teamed up with the pop group The Roots, experience designers Bompas and Parr, and author Spence to create a special music video in 2016 as part of Le Savoir, a multisensory entertainment platform (Birkner, 2016). In this case, the idea was that people sitting at home might enjoy a drink of the beer with a paired music video. Simply moving the cursor while watching the specially composed track and associated music video (called “Sweet to the Bitter End”) allowed the consumer to bring out a sweeter (fruitier) or more bitter version of the instrumentation/video backdrop (cf. the Propel activation in the “Isotonic Drinks” section). The suggestion that this personalized version of sonic seasoning could then be used to adjust the drink to taste – a very literal form of digital seasoning. Importantly, the online activation was linked to a series of experiential dinners. Todd Allen, VP of Global Marketing at Stella Artois reported that: “It’s bringing millennials’ passion points of food, music and art together under one platform to deliver an immersive dining experience, all perfectly paired with Stella Artois…We’re very excited to bring it to the market.” (quoted in Birkner, 2016).

Similarly, the ‘‘Beck’s Frequency’’ activation (see ‘‘Section Beer’’) included an online component where visitors to the website could select a music track and move a curser either right or left, switching between lower-frequency/higher-frequency versions of the selected track.^33^ Consumers were invited to experience the impact of the sonic seasoning as they drank the beer and moved the cursor. In addition, profiles of the DJs who created the tracks for the activation were featured. Jägermeister’s ‘‘Taste Remastered’’ (see Section ‘‘Spirits and liquors’’) also included different offline and online components. The main component of this campaign was the digital interface provided via campaign website. As a promotional support, the campaign was launched by means of physical multisensory tasting events conducted across different European countries, with local influencers. At the end of these events, such influencers were encouraged to take sips of Jägermeister while listening to each of the four sonic seasonings used in the activation, disseminating the campaign via social networks.^34^

Tying music releases to multisensory experiential activations can also benefit the bands, artists, and musicians involved in the projects. The music track created by the Roots for Stella Artois was released on YouTube (Side A/Bitter^35^; Side B/Sweet)^36^, with the release timed to coincide with the previously mentioned Sensorium (Shea, 2017). In 2016, The Brussels Beer Project, launched a beer inspired by the visual and musical identity of the latest album of the rock band “The Editors,” and complemented its promotion by offering their clients an opportunity to participate in a multisensory tasting event (McEachran and O’Mahony, 2016; Reinoso Carvalho et al., 2016a).

## Discussion and Conclusion

The last decade has seen an explosion of interest in sonic seasoning from academics, practitioners (be they marketers, strategists, DJs, sound designers, composers, etc.; see ‘How sound affects our sense of taste’, 2016), the press, and public alike. While sonic seasoning was first documented in the laboratory by scientists (Holt-Hansen, 1968, 1976; Rudmin and Cappelli, 1983), many of the activations that have emerged in recent years (i.e., over the last decade) have been driven by marketing agencies working on behalf of food and beverage companies together with academics interested in crossmodal correspondences/sonic seasoning (see Table 2 for a summary of brand-related activations).

### Emerging Research Directions in Sonic Seasoning

In the coming years, we are likely to see the emergence of generic sensory apps designed to offer multisensory experiences to the masses, with one such service already promising to provide matching sonic seasonings simply by scanning the label of a wine bottle^37^ (see also Werner, 2015; and Baral, 2015, for an earlier branded sensory app).

It is notable that the majority of sonic seasonings used in activations today are limited to instrumental music tracks or soundscapes, devoid of any vocal component (again see Table 2 for a summary). Researchers have, though, now started to assess crossmodal correspondences between tastes and vocal qualities (Simner et al., 2010; Motoki et al., 2019). What is more, there are reasons to believe that vocal utterances and taste qualities might be especially closely linked (Spence, 2012). The use of vocal content could allow for the possibility of semantic priming, as in The Roots track created for a Stella Artois activation called “Sweet ‘til the Bitter End.” The principles of sonic seasoning are likely to increasingly make their way into advertising (see Lowe and Haws, 2017; Lowe et al., 2018; Motoki et al., 2019).

Using sound to influence the mouthfeel properties of food and drink also looks likely to be one of the emerging trends. After all, we rely on sound much more to assess food texture as compared to flavour. The “Smooth Operator” chocolate study demonstrates that sonic seasoning related to food texture are not just built on sound effects or noises associated with eating, but can also be communicated through background music (Reinoso Carvalho et al., 2017; see also Crisinel and Spence, 2011).

While music can undoubtedly be created to match the specific temporal evolution of the flavour of a particular food or beverage product, this is clearly a niche undertaking, with the cost of engaging in such a creative challenge a potential barrier to the future widespread optimization of music with specific flavour experiences. As a result, more basic sonic seasoning examples, such as those mentioned above, with the accentuation of more basic taste/flavour profiles of sweetness, bitterness, and/or saltiness, may be expected to be the most commonly encountered examples of flavour-music pairing in the years ahead (Crisinel et al., 2012; Blecken, 2017).

Given that taste-masking occurs between basic tastes (Breslin and Beauchamp, 1997), it might also be interesting in the future to pursue further whether this is also documented crossmodally, though the evidence to date suggests that crossmodal sensory masking is unlikely to occur (see Spence, 2014). That said, there is another interesting multisensory phenomenon whereby the presentation of the appropriate sound can prevent a visual stimulus from disappearing from awareness (e.g., Adam and Noppeney, 2014; Rodríguez et al., 2021; Sheth and Shimojo, 2014). Similarly, the presence of a sweet taste has been shown to do something very similar for the minty flavour of chewing gum (Davidson et al., 1999). As such, it would be intriguing to know whether sonic sweetness can play the same role in helping to prolong the multisensory flavour experiences and help to prevent habituation as these various interactions between the chemical senses have been shown to do.

Moving forward, an intriguing alternative approach to developing sonic seasoning solutions that may benefit from sensation transference could involve selecting tracks from an individual consumer’s personal playlists (Reinoso-Carvalho et al., 2019; Spence, 2021b). Alternatively, one might consider a “TastEQ” feature in an application that could tweak the frequency and pitch of selected tracks to enhance the relevant taste (raising the pitch/frequency to add sweetness, or lowering it for those who want a little more bitterness, similar to the Beck’s Frequency case presented earlier). There is also the opportunity to go beyond auditory only stimulation, using audiovisual digital activations that may allow for more pronounced sensory manipulation (e.g., see Wang and Spence, 2015b; McGregor, 2017), perhaps even paring meals with shows on digital channels such as Netflix or Amazon Prime (as discussed briefly in Spence et al., 2021).

Furthermore, pursuing the seasoning metaphor, one might also want to develop sonic seasoning to deliver the same effect as adding salt or black pepper to a dish, given its ubiquity as a seasoning on dining tables in many countries. Developing sonic seasoning to mimic the effect of adding soy sauce to a dish would be another interesting direction to pursue, with its combination of salty and umami notes, and once again, the popularity of this fermented seasoning (Haagen-Smit, 1952), especially in the Far East. Another usage case with potential would be to develop minty sonic seasoning to accompany brushing one’s teeth (and so extending sonic seasoning into the oral care space).

As the case studies reviewed in this manuscript make clear, there are several ways to commercialize sonic seasoning design and activation, from the research and development of bespoke, crossmodally congruent music and soundscapes, to selecting pre-existing music tracks that have the requisite sonic properties (be it in terms of their semantic or crossmodally corresponding properties). Nowadays, such curated playlists are often presented via online music platforms such as Deezer, Spotify, or increasingly, Pandora (see Khale, 2018; Spence et al., 2021; Wang et al., 2021a; see also Samuely, 2021, for a Munchery playlist offered by Google Play; and Zarczynski, 2020). Such pairings may even take place as a part of experiential multisensory tasting events (Campo et al., 2021). Indeed, if anything, many activations would appear to becoming increasingly multisensory (e.g., Benjamin, 2016; Ellis, 2017; Glenday, 2017; Hills-Duty, 2017; Abend, 2019; Edwards, 2021; Kulal, 2021).

### On the Challenges of Commercializing Sonic Seasoning

Finally, a note of caution should be offered as these kinds of multisensory events become more prevalent, and brands and advertisers seek new ways of connecting with their customers through the commercialization of sonic seasoning. While the blending of art/design and science is the hallmark of many of the best activations/interventions, it undoubtedly requires a delicate balance between these two disciplines. There is always the danger that, in the pursuit of the performance, the scientific insights and underpinnings may be lost in the creative process. This would be an especially unfortunate outcome for the field, given the growing experimental body of research demonstrating both the associations between specific taste, flavours, aromas, and textures with particular musical parameters on the one hand, and the perceptual influence of matching music on tasting experiences on the other (see Spence et al., 2019; Wang et al., 2021a, for reviews of the peer-reviewed academic literature on sonic seasoning).

While there is undoubtedly a place for the curation of idiosyncratic playlists (see Brown’s beer-music pairing events as one such example, see Table 1), and for the synaesthetic route to music selection/creation (as illustrated by the Campo Viejo 2015 experience mentioned earlier; see Knapton, 2015), the approach advocated here by the authors is based much more firmly on the scientific foundations of crossmodal correspondences (see Spence et al., 2019). For those who choose to adopt such an approach, which we firmly believe provides the most robust foundations for sonic seasoning interventions and activations, there can sometimes be a commercial pressure to bypass the pursuit of scientific validation. However, it is important to recognize that when the science is ignored and/or misinterpreted, the resulting sonic seasoning compositions/choices can all-too-easily end up being haphazard, underwhelming, and perhaps even confusing to the target audience (for example, consider Chihou’s, 2017, review of Macallan Concept Number 2). Such a result is not only counterproductive for brands, but also for the consumer perception of crossmodal science as well (especially when the scientific aspect is purportedly emphasized in many brand communications).

Even when a creative approach to sonic seasonings takes the scientific insights to heart, such as the menu of sonic properties that have been scientifically shown to correspond to a particular desired taste quality, the ensuing composition may nevertheless lose (or gain) something in translation (see Crisinel et al., 2013, discussed earlier for at least one occasion where this appears to have occurred). Given that, where possible, scientifically validating the crossmodal musical compositions/selections is to be desired, it is striking how few of the commercial examples discussed in Section “Case studies of sonic seasoning: At the nexus of art, science, and marketing” involved any kind of empirical testing of the created compositions effectiveness in modulating taste/flavour in the manner anticipated. While not every experiential activation involving the use of sonic seasonings need to involve rigorous empirical validation, we would strongly advocate for the involvement of crossmodal scientists working alongside brand and agency collaborators while the experiences are developed (see Velasco et al., 2013; Spence et al., 2014, for a few examples where validation did take place). Striking the right balance between art/design and science, amidst commercial constraints and time-sensitive activations, is just part of what makes working in this area such an engaging and exciting challenge for commercially minded scientists. Caveats aside, it’s clear that brands can use sonic seasonings to create innovative ways to connect with their consumers in ways that can, quite literally, “sound delicious.”

## Author Contributions

All authors contributed to the writing of this review, and approved the final draft.

## Conflict of Interest

SK works for SiriusXM, which owns Pandora. The various commercial activations on which the various authors worked (typically as paid consultants) are all clearly identified in the text. The remaining authors declare that the research was conducted in the absence of any commercial or financial relationships that could be construed as a potential conflict of interest.

## Publisher’s Note

All claims expressed in this article are solely those of the authors and do not necessarily represent those of their affiliated organizations, or those of the publisher, the editors and the reviewers. Any product that may be evaluated in this article, or claim that may be made by its manufacturer, is not guaranteed or endorsed by the publisher.

## Footnotes

1
https://www.hgem.com/opinion/what-is-sonic-seasoning

2 The term “tafelmusik” has been used since the mid-16^th^ Century to refer to musical compositions that were intended to be played at feasts and banquets (Littler, 1989; Reimer, 1972).

3 https://itunes.apple.com/us/app/haagen-dazs-concerto-timer/id670015815?mt=8; https://vimeo.com/71502987

4
https://www.finnair.com/cn/gb/stevenliu/en

5 With their “Hear the taste” campaign, Finnair created a sound-based food-experience-enhancing app that runs on WeChat (a Chinese messaging app), which aims to allow customers to take pictures of their food and obtain soundscape that will help to enhance their experience.

6
https://www.youtube.com/watch?v=rcZjj3mxboI

7 As Barb Stuckey (2012) notes in her book *Taste what you’re missing*, different artificial sweeteners tend to have very different temporal (intensity) profiles (cf. Sjöström and Cairncross, 1955). In fact, different sweeteners are often combined specifically so as to try and imitate the temporal profile of natural sugar, as much as possible as this is recognized to be an important part of the taste experience. As such, it would be intriguing to know to what extent the “roundness” typically associated with sweetness is linked to the gradual intensity changes over time for sucrose versus other artificial sweeteners.

8 https://www.krug.com/playlist/krug-echoes; https://www.krug.com/krug-lovers/ozark-henry

9
https://www.courvoisier.com/global/experience/

10
https://kitchen-theory.com/portfolio-item/5-generations-blending-excellence-chivas-ultis/

11
https://youtu.be/CxPNOMmrOJs

12
http://maximegoulet.com/symphonic-chocolates/

13
https://youtu.be/UA_uGJ5vjWg

14
https://www.youtube.com/watch?v=rph6oyIEJ9o

15
https://www.youtube.com/watch?v=wvH_fYCoPzs

16
http://ivaudiobranding.com/portfolio_cadbury.php

17
https://soundcloud.com/user-181909589

18
https://www.mullenlowegroup.com/news/magnums-pleasure-sensorium/

19
https://www.designmynight.com/london/whats-on/food-drink/magnum-pleasure-sensorium

20
https://www.lenez.com/es/editions-jean-lenoir/prensa/caf%C3%A9

21
https://open.spotify.com/user/avvwt825xiyroz9mc3lcvgyu4

22
https://youtu.be/FHec_uFx4x8

23
https://youtu.be/UODZMzq7UtA

24
https://www.castellocheese.com/en-us/feed-your-senses/

25
http://www.gq.com.mx/lujo-gq-elyx/articulos/maridaje-musical-sonic-seasoning-con-deezer-elyx-escondite/4286

26
https://nmbx.newmusicusa.org/food-opera-merging-taste-and-sound-in-real-time/

27
https://www.mugaritz.com/en/music-and-gastronomy-at-the-service-of-technology/co-1613047208/

28
https://youtu.be/t93YpTapnXQ

29
http://ivaudiobranding.com/soundsdelicious/

30
https://kitchen-theory.com/valentinesday/

31
https://kitchen-theory.com/mothers-day-afternoon-tea/

32
https://www.exploretock.com/playlists/

33
https://www.beckscerveja.com.br/frequenciabecks/en/

34
https://www.jagermeister.com/de-DE/taste-remastered

35
https://youtu.be/YUo0-UiKgVE

36
https://youtu.be/w2VVU_QWlzE

37
http://winelistening.com/
